# The Expression of von Willebrand Factor-Binding Protein Determines Joint-Invading Capacity of Staphylococcus aureus, a Core Mechanism of Septic Arthritis

**DOI:** 10.1128/mBio.02472-20

**Published:** 2020-11-17

**Authors:** Manli Na, Zhicheng Hu, Majd Mohammad, Mariana do Nascimento Stroparo, Abukar Ali, Ying Fei, Anders Jarneborn, Peter Verhamme, Olaf Schneewind, Dominique Missiakas, Tao Jin

**Affiliations:** a Department of Rheumatology and Inflammation Research, Institution of Medicine, Sahlgrenska Academy at University of Gothenburg, Gothenburg, Sweden; b Department of Microbiology and Immunology, The Affiliated Hospital of Guizhou Medical University, Guiyang, China; c Center for Molecular and Vascular Biology, Department of Cardiovascular Sciences, University of Leuven, Leuven, Belgium; d Department of Microbiology, University of Chicago, Chicago, Illinois, USA; e Department of Rheumatology, Sahlgrenska University Hospital, Gothenburg, Sweden; New York University School of Medicine

**Keywords:** von Willebrand factor-binding protein, von Willebrand factor, *Staphylococcus aureus*, septic arthritis, mouse

## Abstract

Septic arthritis, one of the most dangerous joint diseases, is predominantly caused by Staphylococcus aureus. In contrast, coagulase-negative staphylococci are rarely found in septic arthritis. We hypothesize that coagulases released by S. aureus, including coagulase (Coa) and von Willebrand factor-binding protein (vWbp), play potent roles in the induction of septic arthritis. Four isogenic S. aureus strains differing in expression of coagulases (wild-type [WT] Newman, Δ*coa*, Δ*vwb*, and Δ*coa* Δ*vwb*) were used to induce septic arthritis in both wild-type and von Willebrand factor (vWF)-deficient mice. Septic arthritis severity was greatly reduced when wild-type mice were infected with the Δ*coa* Δ*vwb* and Δ*vwb* variants compared to WT or Δ*coa* strains, suggesting that vWbp rather than Coa is a major virulence factor in S. aureus septic arthritis. vWF-deficient mice were more susceptible to bone damage in septic arthritis, especially when the Δ*vwb* strain was used. Importantly, no difference in arthritis severity between the Δ*vwb* and WT strains was observed in vWF-deficient mice. Collectively, we conclude that vWbp production by S. aureus enhances staphylococcal septic arthritis.

## Abstract (precis)

Septic arthritis remains one of the most dangerous joint diseases with a rapidly progressive disease character. Despite advances in the use of antibiotics, permanent reductions in joint function due to joint deformation and deleterious contractures occur in up to 50% of patients with septic arthritis. So far, it is still largely unknown how S. aureus initiates and establishes joint infection. Here, we demonstrate that von Willebrand factor-binding protein expressed by S. aureus facilitates the initiation of septic arthritis. Such effect might be mediated through its interaction with a host factor (von Willebrand factor). Our finding contributes significantly to the full understanding of septic arthritis etiology and will pave the way for new therapeutic modalities for this devastating disease.

## INTRODUCTION

Septic arthritis, also known as infectious arthritis or joint infection, remains one of the most dangerous joint diseases with a rapidly progressive disease character. Staphylococcus aureus is the most common pathogen of septic arthritis. Despite advances in the use of antibiotics, permanent reductions in joint function due to joint deformation and deleterious contractures occur in up to 50% of patients with septic arthritis (1). Additional challenge is posed by increasing antibiotic resistance of S. aureus (2). Our recent work suggests that the combination of antibiotics and biological drugs targeting inflammatory mediators is able to minimize postinfectious sequelae caused by long-lasting joint inflammation (3, 4). However, there are potential dangers associated with the combination therapy of antibiotics and biologics in septic arthritis (3, 5). Understanding how S. aureus initiates and establishes joint infection would allow the identification of therapeutic targets beyond those that reduce inflammatory mediators.

Bacterial joint invasion is the key step for triggering septic arthritis, and in most cases, the initial cause of disease is invading S. aureus bacteria in affected joints. Hematogenous spread of S. aureus to the joint cavity is the most common reported route of acquiring septic arthritis (6). Defects in the host’s innate immunity exacerbate the susceptibility toward S. aureus in septic arthritis, which is correlated with impaired bacterial clearance (7, 8). However, it is still largely unknown which factors determine the joint-invading process of S. aureus. Unlike S. aureus, the related coagulase-negative staphylococci (CoNS) are hardly found in native septic arthritis, suggesting coagulases might play a major role in disease pathogenesis.

S. aureus has a marked propensity to activate both the coagulation and fibrinolytic systems (9). Coagulation is promoted by two secreted enzymes (coagulases), coagulase (Coa) and von Willebrand factor-binding protein (vWbp), that activate host prothrombin and consequently cleave fibrinogen to fibrin (10). Both Coa and vWbp promote clotting of soluble fibrinogen, plasma, or blood by forming a stable complex with prothrombin. Apart from its coagulation-promoting property, von Willebrand-binding protein has also high affinity to von Willebrand factor that can form ultralarge multimers retained on the endothelial cell surface upon activation of endothelial cells (11). It has been shown that interaction between von Willebrand factor and vWbp contributes to vascular adhesion of S. aureus (12).

In the present study, we examine the contribution of Coa and vWbp for joint-specific invasiveness by S. aureus in septic arthritis.

## RESULTS

### Septic arthritis is greatly reduced upon infection with S. aureus lacking both Coa and vWbp.

**FIG 1:**
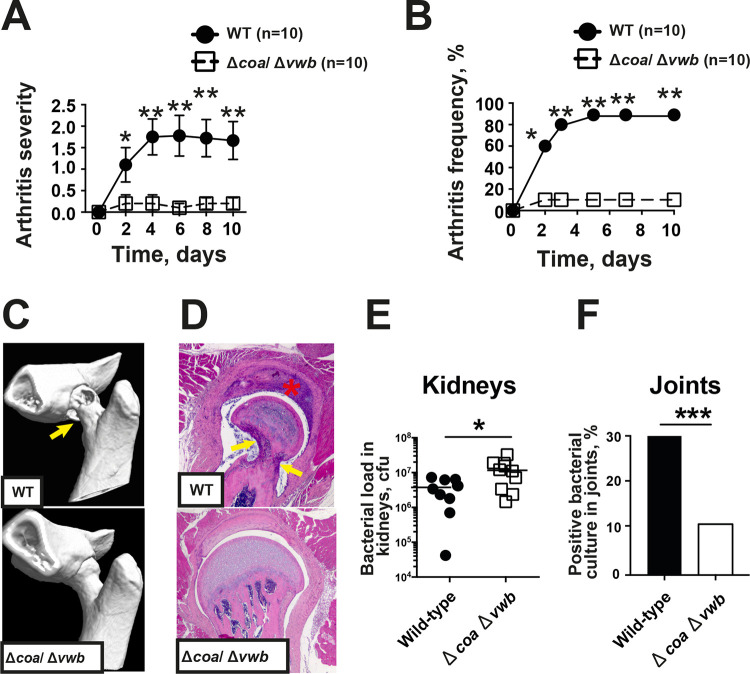
Coagulases are critical virulence factors of S. aureus septic arthritis. NMRI mice inoculated with S. aureus Newman strain or Δ*coa* Δ*vwb* mutant strain (4.0 × 10^6^ CFU/mouse) were sacrificed on day 10. The severity (A) and frequency (B) of clinical arthritis were observed for 10 days postinfection. (C) Representative microcomputed tomography images of an intact hip joint from an NMRI mouse infected with Δ*coa* Δ*vwb* (bottom) and destroyed hip joint from NMRI mice infected with the Newman WT (top). The arrows indicate bone destruction. (D) Representative photomicrographs of histologically intact knee joint from a NMRI mouse infected with Δ*coa* Δ*vwb* (bottom) and a heavily inflamed hip joint with severe bone and cartilage destruction from NMRI mouse with septic arthritis inoculated with the Newman strain (top), stained with hematoxylin and eosin. Original magnification, ×10. The asterisk indicates heavily inflamed synovium and the arrows indicate bone destruction. (E and F) Bacterial loads in kidneys (E) and positive bacterial culture in joints (F) on day 3 were compared between Newman- and Δ*coa* Δ*vwb*-infected mice. Statistical evaluations were performed using the Mann-Whitney *U* test and chi-square test. Data were presented as mean ± SEM. *, *P < *0.05; **, *P < *0.01; ***, *P < *0.001.

To understand the overall role of coagulases in S. aureus-induced septic arthritis, a mutant strain lacking both the Coa and vWbp encoding genes (Δ*coa* Δ*vwb*) was compared with its parental Newman (WT) strain in the septic arthritis mouse model. Infection with the Δ*coa* Δ*vwb* strain resulted in greatly reduced arthritogenicity compared to its parental strain (Fig. 1). The severity of clinical arthritis was attenuated in mice infected with the Δ*coa* Δ*vwb* mutant compared to animals infected with the Newman strain during the whole course of disease (Fig. 1A). The frequency of arthritis in the Δ*coa* Δ*vwb* mutant-infected group was also significantly decreased. On day 2 postinfection, 10% of animals in Δ*coa* Δ*vwb*-infected group developed arthritis, whereas the arthritis frequency in the control group was 60% (Fig. 1B). At the end of the experiment (day 10), the Δ*coa* Δ*vwb*-infected group had an arthritis frequency of 20% compared to 78% in mice infected with the wild-type Newman strain.

The representative computed tomography (CT) images show severe bone destruction in a hip joint from a mouse infected with the wild-type strain Newman (Fig. 1C, top) and a healthy hip joint from a mouse infected with the Δ*coa* Δ*vwb* mutant (Fig. 1C, bottom). Histopathological Fig. 1D shows a septic arthritis hip joint with heavily inflamed synovium and severe bone erosions (top) and a healthy joint with single-layer synovium and intact cartilage (bottom). Histopathologically verified synovitis and the extent of joint destruction were significantly attenuated in Δ*coa* Δ*vwb*-infected mice compared to mice infected with the parental Newman strain.

The joints and kidneys were collected on day 3 postinfection, and the CFU counts were processed by plating serial dilutions of ground tissues. Interestingly, animals infected with the Δ*coa* Δ*vwb* mutant had slightly higher bacterial CFU counts in kidneys than mice infected with the Newman strain, whereas bacteria positive joints were found in 30% of joints from Newman-infected mice and only 11% in Δ*coa* Δ*vwb* mutant-infected mice (Fig. 1E and F), strongly suggesting that S. aureus-producing coagulases have joint-invading propensity.

### vWbp rather than Coa mediates S. aureus-induced septic arthritis.

**FIG 2:**
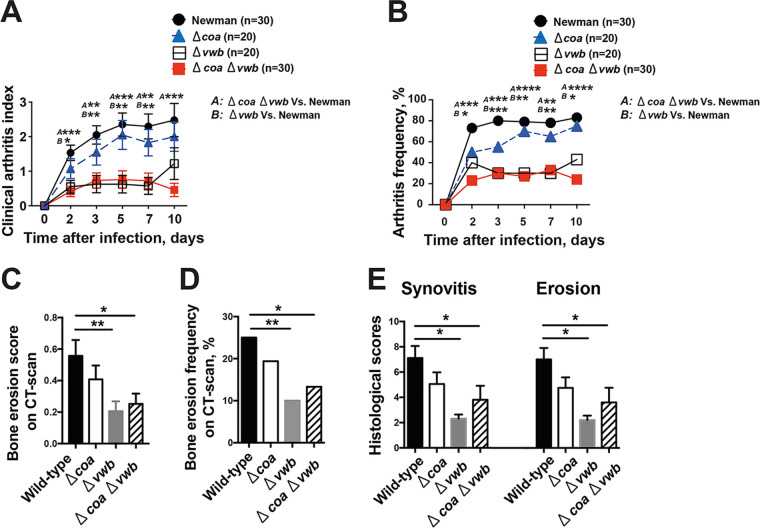
vWbp rather than Coa determines bacterial joint invasiveness. NMRI mice inoculated with S. aureus Newman, Δ*coa*, Δ*vwb*, and Δ*coa* Δ*vwb* strains (4.0 × 10^6^ CFU/mouse) were sacrificed on day 10. The data from 3 independent experiments were pooled. (A and B) Severity (A) and frequency (B) of clinical arthritis were observed for 10 days postinfection. (C and D) Cumulative bone erosion scores (C) and frequency of bone destruction (D) of the joints from all 4 limbs of NMRI mice as assessed by microcomputed tomography scan. (E) Histological evaluation of the joints, including synovitis and bone erosion scores from all 4 limbs 10 days after infection. Statistical evaluations were performed using the Mann–Whitney U test (A, C, and E) and Fisher’s exact test (B and D). Data are expressed as mean values ± SEM. *, *P < *0.05; **, *P < *0.01, ***, *P < *0.001.

To further study the relative importance of Coa and vWbp during joint infections, groups of animals were infected with four isogenic S. aureus strains differing in expression of coagulases (Fig. 2). Compared to mice infected with the Newman (WT) strain, mice infected with both the single Δ*vwb* or double Δ*coa* Δ*vwb* mutants developed significantly milder and less frequent clinical arthritis. This difference was already noticeable on day 2 postinfection and continued until the end of the experiment. In comparison, animals infected with the Δ*coa* mutant developed severe clinical arthritis in a manner indistinguishable from mice infected with Newman (Fig. 2A and B), suggesting vWbp rather than Coa is a crucial virulence factor for induction of septic arthritis. In line with clinical arthritis data, both severity (Fig. 2C) and frequency (Fig. 2D) of bone erosion with micro-CT scan data were significantly decreased in mice infected with either single Δ*vwb* or double Δ*coa* Δ*vwb* mutants compared with animals infected with the wild-type parental or Δ*coa* mutant strains. In fact, the last two groups did not display any difference (Fig. 2C and D). In addition, histopathologically verified synovitis and the extent of joint destruction were also significantly reduced in mice infected with either Δ*vwb* or Δ*coa* Δ*vwb* mutants compared with mice infected with the wild-type parental or Δ*coa* mutant strains (Fig. 2E).

**FIG 3:**
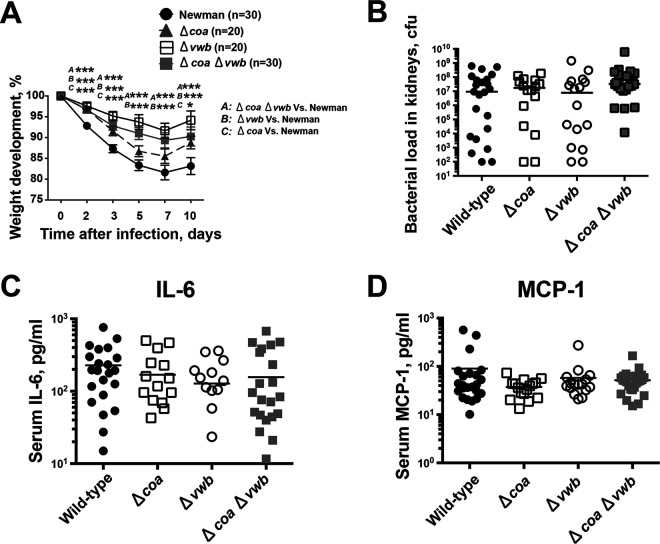
The impact of coagulase expression on weight loss, bacterial clearance, and cytokine production in mouse with septic arthritis. NMRI mice inoculated with S. aureus Newman, Δ*coa*, Δ*vwb*, and Δ*coa* Δ*vwb* strains (4.0 × 10^6^ CFU/mouse) were sacrificed on day 10. The data from 3 independent experiments were pooled. (A) Changes in body weight registered as percentages of the initial body weight. (B to D) Persistence of S. aureus in kidneys (B), serum IL-6 (C), and MCP-1 levels (D) 10 days after infection. Statistical evaluations were performed using the Mann-Whitney U test. Data are expressed as mean values ± SEM. *, *P < *0.05; ***, *P < *0.001.

Animals infected with single Δ*vwb* or double Δ*coa* Δ*vwb* mutants lost significantly less body weight than mice infected with the Newman strain during the whole course of disease (Fig. 3A). Interestingly, loss of *coa* expression also resulted in reduced weight loss compared to infections with wild-type S. aureus on days 2, 3, and 10 (Fig. 3A). On day 10 after infection, all four groups of animals displayed similar bacterial loads in kidney tissues (Fig. 3B) as well as similar serum levels of interleukin-6 (IL-6) (Fig. 3C) and monocyte chemoattractant protein 1 (MCP-1) (Fig. 3D).

### vWbp-deficient S. aureus caused as severe bone erosion as its parental strain in vWF-deficient mice.

**FIG 4:**
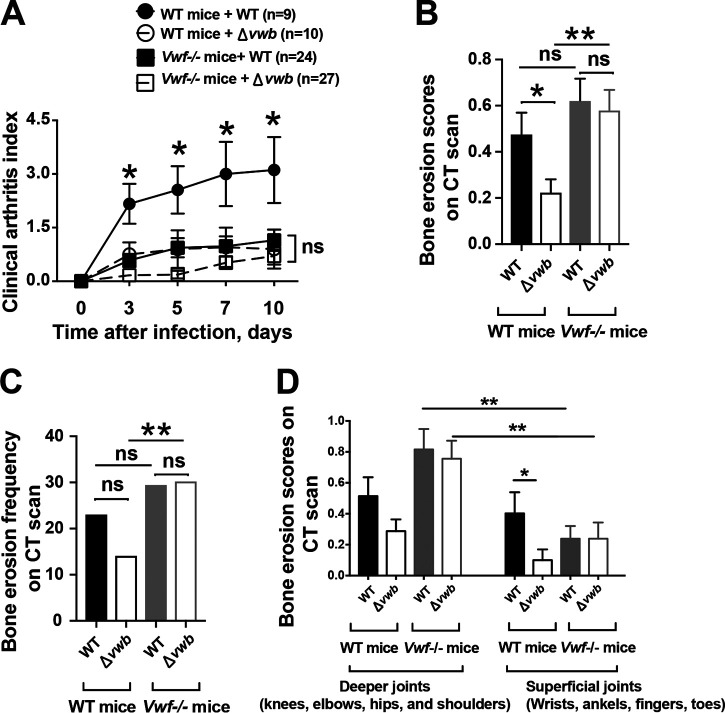
vWbp-deficient S. aureus caused as severe bone erosion as its parental strain in vWF-deficient mice. C57BL/6 and *Vwf*^−/−^ C57BL/6 mice inoculated with S. aureus Newman strain and *ΔvWbp* mutant strain (4.0 × 10^6^ CFU/mouse) were sacrificed on day 10. The data from 3 independent experiments were pooled. (A) Severity of clinical arthritis in the mice was observed for 10 days postinfection. (B and C) Cumulative bone erosion scores (B) and frequency of bone destruction (C) of the joints from all 4 limbs of mice as assessed by microcomputed tomography scan. The joints were divided to deeper joints (knees, elbows, hips, and shoulders) and superficial joints (wrists, ankles, fingers, and toes). (D) Involvement of septic arthritis in deeper and superficial joints was then compared. Statistical evaluations were performed using the Mann-Whitney U test (A, B, and D) and chi-square test (C). Data are expressed as mean values ± SEM. ns, not significant; *, *P < *0.05; **, *P* < 0.01.

Both Coa and vWbp promote blood coagulation. However, only vWbp interacts with von Willebrand factor (vWF) that forms ultralarge multimers retained on the endothelial cell surface upon release from these cells (11). To evaluate the contribution of this interaction in our disease model, vWF-deficient and C57BL/6 WT mice were infected with the Δ*vwb* mutant and the parental strain. In agreement with data obtained from NMRI mice, C57BL/6 WT mice infected with Δ*vwb* displayed significantly milder clinical arthritis than WT mice infected with the WT Newman strain. Relatively mild clinical arthritis was observed in vWF-deficient mice infected with either Δ*vwb* or Newman strains, and no difference was found between these two groups (Fig. 4A). Results from micro-CT scan confirmed our clinical observation. As expected, significantly milder and less frequent bone destruction was found in WT mice inoculated with the Δ*vwb* mutant compared to infection with the Newman strain. However, no difference could be detected when vWF-deficient mice were used for infection with either Δ*vwb* or Newman (Fig. 4B and C).

Surprisingly, a discrepancy was observed between clinical arthritis evaluation and micro-CT scan results in vWF-deficient mice, as bone destruction was severe, but clinical arthritis was relatively mild in those mice. As arthritis of deeper joints (knees, elbows, hips, and shoulders) was assessable by micro-CT scan but not by clinical evaluation, we hypothesized that vWF-deficient mice had more septic arthritis in deeper joints than superficial joints. A more detailed subgroup analysis was performed to investigate the reason for this discrepancy (Fig. 4D). Indeed, both Δ*vwb* mutant and Newman strains displayed invading propensities to deeper joints in vWF-deficient mice. Such joint preference of S. aureus was not observed with C57BL/6 wild-type mice.

### vWbp deficiency had no impact on weight development and kidney bacterial load in vWF-deficient mice.

**FIG 5:**
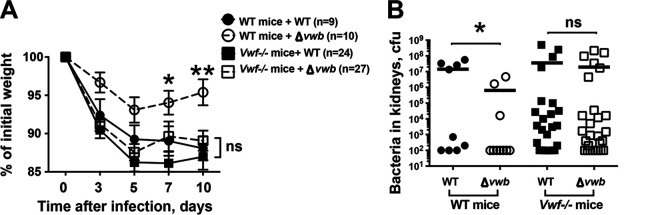
vWbp deficiency had no impact on weight development and kidney bacterial load in vWF-deficient mice.C57BL/6 and *Vwf*^−/−^ C57BL/6 mice inoculated with S. aureus Newman and Δ*vwb* mutant strains (4.0 × 10^6^ CFU/mouse) were sacrificed on day 10. The data from 3 independent experiments were pooled. (A) Changes in body weight registered as percentages of the initial body weight. (B) Persistence of S. aureus in kidneys 10 days after infection. Statistical evaluations were performed using the Mann-Whitney U test. Data are expressed as mean values ± SEM. ns, not significant; *, *P < *0.05; **, *P < *0.01.

In line with the data from NMRI mice, C57BL/6 WT mice infected with the Δ*vwb* mutant had better weight maintenance than animals infected with the Newman strain. In contrast, infection with both the Δ*vwb* mutant and Newman strain resulted in gradual weight loss in vWF-deficient mice, and no tangible difference was found between these two groups (Fig. 5A). With respect to bacterial dissemination to kidneys, C57BL/6 WT mice infected with the Δ*vwb* mutant harbored significantly lower CFU counts than animals infected with the Newman strain. Interestingly, counts remained high for both infections in vWF-deficient mice (Fig. 5B).

## DISCUSSION

Coagulase-negative staphylococci hardly cause any hematogenous septic arthritis in native joints. S. aureus, another species expressing coagulases in the very same genus group, is, however, the top culprit responsible for the disease (13). Despite the fact that coagulases have been shown to be implicated in different types of S. aureus infections (10, 12, 14), much is still unknown about the role of coagulases in S. aureus septic arthritis. In the current study, we show for the first time that vWbp rather than Coa is a critical S. aureus virulence factor that mediates the bacterial joint-invading capacity in septic arthritis. The joint propensity of S. aureus might rely on the interaction between S. aureus vWbp and the host factor vWF.

Several surface proteins known as adhesins have been previously reported to contribute to S. aureus septic arthritis (15). The clumping factor A (ClfA) is known to be one of those virulence factors, and the virulence is not mediated through the interaction between clumping factor A and fibrinogen (16, 17). vWbp, a secreted protein, however, can interact with ClfA on the bacterial surface after secretion, mediating adhesion of S. aureus to vWF and vascular endothelium under shear stress (18). We speculate that bacterial vascular adhesion mediated by a ClfA-vWbp-vWF complex might be one of the explanations for the pathogenic role of ClfA in septic arthritis. Of note, protein A, also possessing binding capacity to vWF (19), has also been shown to be a virulence factor in S. aureus septic arthritis (20). However, under conditions of high shear stress, protein A does not promote bacterial adherence to vWF (21), which rules out the possibility of bacterial vascular adhesion mediated by protein A in septic arthritis.

vWbp displays species-specific activity as a coagulase. It coagulates human and porcine plasma efficiently but has modest coagulating activity in mouse plasma (22), whereas Coa is an efficient coagulase in mouse plasma. Thus, vWbp activity in the mouse appears to be largely mediated via interaction with vWF and, to a smaller degree, to coagulation. It has been shown that both Coa and vWbp are required for abscess formation (10) and *in vivo* bacteria adhesion to blood vessel wall (12). In contrast, in our septic arthritis model, the Coa-deficient strain displayed similar or slightly lower virulence than the parental strain, suggesting that Coa-mediated fibrin formation does not play a major role in the pathogenesis of septic arthritis. Yet the successful attachment of bacteria to the blood vessel walls, mediated by the vWbp-vWF interaction, may be enhanced by Coa release and local fibrin-forming activity, which will recruit platelets to bacterial microthrombi. Indeed, the Coa-fibrin scaffold shields S. aureus from opsonophagocytosis and innate immune attack (23). In humans, since vWbp possesses both coagulase- and vWF-binding properties, the importance of vWbp in pathogenesis of septic arthritis might be even greater than in the mouse model.

The second top-ranked bacteria causing septic arthritis are *Streptococcus* spp. (13). Some species of streptococci, such as S. pneumoniae, are known to express surface-exposed enolase as vWF-binding protein, which mediates bacteria anchoring within the bloodstream and promotes intravascular bacterial aggregation (24). Other arthritogenic bacteria species also express vWbp-like proteins. For example, Staphylococcus lugdunensis is known to express vWF-binding protein (25) that mediates bacterial adhesion to the cardiac valves and initiates endocarditis (26). Thus, the vWF-binding protein/vWF-mediated bacteria joint-invading capacity might be a universal disease mechanism for hematogenous septic arthritis.

Preexisting joint disorders, such as rheumatoid arthritis (RA), are associated with increased risk for septic arthritis (27). Release of proinflammatory cytokines, including tumor necrosis factor (TNF) and IL-6 in synovial local joints, is one of the hallmarks in the pathogenesis of RA. Inhibitors for those proinflammatory cytokines have achieved the most notable clinical success in the treatment of RA (28). Interestingly, IL-8 and TNF stimulate release of ultralarge and hyperreactive vWF by endothelial cells. Simultaneously, IL-6 inhibits vWF cleavage by ADAMTS13 under flowing conditions (29). This results in the accumulation of vWF on the surface of vascular endothelia of inflamed joints, which provides a perfect anchoring base for bacteria producing proteins such as vWbp. Initial seeding of bacteria on the blood vessel wall in the joints permits proliferation and disease. A similar disease mechanism has been demonstrated in an S. aureus endocarditis model, as the local inflammation of cardiac valves causes endothelial activation and vWF release, which recruits platelet accumulation and captures S. aureus to the valve surface (30). Indeed, patients with RA display elevated levels of vWF, which correlate positively with inflammatory markers (31). The increased risk for septic arthritis in RA patients might be partially explained by endothelial injury and accumulation of hyperreactive vWF on the endothelium surface of inflamed joints. Future studies are warranted to understand how inflammation impacts the hemostatic status in joints, bacterial seeding to the local blood vessel, and development of septic arthritis.

It is clear that vWF-deficient mice are more susceptible to the bone damages in S. aureus septic arthritis than the wild-type mice, especially in the bacteria lacking vWbp expression. This suggests that host vWF plays a protective role, especially for the bacteria strains that do not express vWbp. The protective role of vWF in septic arthritis might be explained by the link between vWF and inflammation, as vWF is known to possess the capacity to attract the leukocytes by either direct binding or recruitment of platelets, which, in turn, recruit leukocytes (32). The influx of innate immune cells into infected joints is crucial for bacteria elimination and better outcomes in septic arthritis (33). However, it still remains elusive why the protective role of vWF is most predominant when vWbp-deficient bacteria were invading.

Another unexpected finding was that the characteristics of septic arthritis are somehow different in the vWF-deficient mice compared to WT mice, as there was significantly deeper joint involvement in the vWF knockout mouse than WT controls. The question then arises, is there any better way to confirm the relevance of vWbp-vWF interaction in septic arthritis? The binding of vWbp and vWF is specific and mediated by a region of 26-amino-acid (aa) residues in the C-terminal part of vWbp (34). To generate an S. aureus mutant strain expressing vWbp with the vWF-binding site disrupted and use it in our septic arthritis model will ultimately determine the importance of vWbp-vWF interaction in septic arthritis in the future. Indeed, a similar strategy has been successfully applied before to study the role of fibrinogen-binding sites of clumping factor A in S. aureus infections (35, 36).

Here, we have demonstrated that vWbp determines S. aureus joint-invading capacity in hematogenous septic arthritis. This knowledge contributes significantly to our full understanding of septic arthritis etiology and will pave the way for fundamentally new therapeutic modalities for this devastating disease.

## MATERIALS AND METHODS

### Mice.

Female NMRI mice and C57BL/6 wild-type mice, aged 6 to 8 weeks, were purchased from Envigo (Venray, Netherlands) and Charles River Laboratories (Sulzfeld, Germany), respectively. Homozygous *Vwf*^−/−^ C57BL/6 mice (12) were bred in the animal facility of the Department of Rheumatology and Inflammation Research, University of Gothenburg. Mice were kept under standard conditions of temperature and light and were fed laboratory chow and water *ad libitum*. The Ethics Committee of Animal Research of Gothenburg approved the study, and animal experimentation guidelines were strictly followed.

### Preparation of bacterial strains.

S. aureus Newman wild-type strain and isogenic deletion strains Δ*coa*, Δ*vwb*, and Δ*coa* Δ*vwb* (10) were cultured on blood agar plates for 24 h, harvested, and kept frozen at −20°C in phosphate-buffered saline (PBS) containing 5% bovine serum albumin (BSA) and 10% dimethyl sulfoxide (DMSO). Before the experiments, the bacterial solutions were thawed, washed with sterile PBS, and adjusted to the required concentration.

### Mouse model for hematogenous S. aureus arthritis.

We used a well-established mouse model of septic arthritis that closely resembles human infectious arthritis spread hematogenously (37). Briefly, mice were inoculated intravenously (i.v.) via the tail vein with 0.2 ml of S. aureus suspension (2.0 × 10^7^ to 2.5 × 10^7^ CFU/ml). Animals were weighed regularly and examined for arthritis by observers blinded to the different groups (M.N. and T.J.). Weight loss and clinical signs of septic arthritis were followed for up to 10 days (days 3, 5, 7, and 10). Blind observers visually inspected all 4 paws of each mouse (hands and feet). Arthritis was defined as erythema and swelling of the joints. To assess the severity of arthritis, a clinical scoring system ranging from 0 to 3 was used as previously described (7). After sacrificing the mice at day 10, the kidneys were obtained for assessment of bacterial dissemination and persistence in organ tissues, serum samples were collected to assess cytokine levels, and the paws were obtained for radiological examination of bone erosions followed by microscopic evaluation of synovitis and destruction of cartilage and bone.

In total, 7 independent *in vivo* experiments were performed. To understand the overall role of coagulases in septic arthritis, NMRI mice (*n* = 10/group) were inoculated with S. aureus Newman strain or the Δ*coa* Δ*vwb* mutant strain (experiment 1). To elucidate the differential role of coagulases in septic arthritis, NMRI mice (*n* = 20 to 30/group) were inoculated with S. aureus Newman, Δ*coa*, Δ*vwb*, and Δ*coa* Δ*vwb* strains (experiments 2 to 4). To study the impact of the vWbp-vWF interaction in the development of septic arthritis, *Vwf*^−/−^ mice and wild-type mice were infected with either S. aureus Newman or Δ*vwb* strains (*n* = 9 to 27/group, experiments 5 to 7).

### Serum cytokine measurements.

Blood collected from animals in arthritis experiments was centrifuged for 15 min at 13,200 rpm after clot formation. Serum was removed and stored at −20°C until analysis. The levels of IL-6 and MCP-1 in serum were quantified using DuoSet enzyme-linked immunosorbent assay (ELISA) kits (R&D Systems, Abingdon, UK).

### Bacteriologic examination in kidneys and joints.

Kidneys were aseptically removed after the mice had been sacrificed. Kidneys were visually examined and blindly judged by two observers (M.N. and A.A.) for degree of abscess formation. A scoring system of 0 to 3 was used as previously described (7). The kidneys were homogenized, serially diluted in PBS, and transferred to agar plates containing 5% horse blood. The bacteria were cultured for 24 h at 37°C and quantified in CFU.

All 4 limbs were divided into 12 joint groups per mouse (hands, elbows, shoulders, feet, knees, and hips) and separately homogenized with a TissueLyser (Qiagen), diluted in PBS, spread on horse blood agar plates, and incubated for 24 h at 37°C. The threshold of positivity for bacteria in the joint was set as equal or more than 10 CFU/joint.

### Microcomputed tomography.

The joints were fixed in 4% formaldehyde for 3 days and then transferred to PBS. Afterward, all 4 limbs were scanned and reconstructed into a three-dimensional structure with SkyScan 1176 micro-CT (Bruker, Antwerp, Belgium) with the settings adjusted to a voxel size of 35 μm with a 0.2-mm aluminum filter at 45 kV/455 μA. The X-ray projections were obtained at 0.7° intervals with a scanning angular rotation of 180°. The projection images were reconstructed into three-dimensional images using NRecon software (version 1.6.9.8; Bruker) and analyzed with CT Analyzer (version 2.7.0; Bruker). After reconstruction, experienced observers (M.N. and Y.F.) evaluated, in a blinded manner, the extent of bone and cartilage destruction on a grading scale from 0 to 3 as previously described (38).

### Histopathological examination.

After scanning, the joints were decalcified, embedded in paraffin, and sectioned with a microtome. Tissue sections were thereafter stained with hematoxylin and eosin. All the slides were coded and assessed under a microscope in a blinded manner by two observers (M.N. and T.J.) with regard to the degree of synovitis and cartilage-bone destruction as previously described (5).

### Statistical analysis.

Statistical significance was assessed using the Mann-Whitney U test, Fischer's exact test, and Mantel-Cox log-rank test as appropriate. Results are reported as the mean ± standard error of the mean (SEM) unless indicated otherwise. A *P* value of <0.05 was considered statistically significant. Calculations were performed using GraphPad Prism version 7.0b software for Mac (GraphPad Software, La Jolla, CA, USA).
